# Long-Term Recordings of Multiple, Single-Neurons for Clinical Applications: The Emerging Role of the Bioactive Microelectrode

**DOI:** 10.3390/ma2041762

**Published:** 2009-11-05

**Authors:** Karen A. Moxon, Stefanie Hallman, Aswin Sundarakrishnan, Margaret Wheatley, Jonathan Nissanov, Kenneth A. Barbee

**Affiliations:** 1 Drexel University, School of Biomedical Engineering, Science and Health Systems, 3141 Chestnut Street Philadelphia, PA 19104, USA; E-Mails: stefanie.hallman@gmail.com (S.H.); wheatley@coe.drexel.edu (M.W.); jonathan.nissanov@gmail.com (J.N.); aswin.sundar@gmail.com (A.S.); kab33@drexel.edu (K.A.B.); 2 Drexel University, College of Medicine, Department of Neurobiology, 2900 Queen Lane, Philadelphia, PA 19129, USA

**Keywords:** brain-machine interface, immunohistochemistry, multiple-single, neuron, electrophysiology, glial scar, microelectrodes

## Abstract

In 1999 we reported an important demonstration of a working brain-machine interface (BMI), in which recordings from multiple, single neurons in sensorimotor cortical areas of rats were used to directly control a robotic arm to retrieve a water reward. Subsequent studies in monkeys, using a similar approach, demonstrated that primates can use a BMI device to control a cursor on a computer screen and a robotic arm. Recent studies in humans with spinal cord injuries have shown that recordings from multiple, single neurons can be used by the patient to control the cursor on a computer screen. The promise is that one day it will be possible to use these control signals from neurons to re-activate the patient’s own limbs. However, the ability to record from large populations of single neurons for long periods of time has been hampered because either the electrode itself fails or the immunological response in the tissue surrounding the microelectrode produces a glial scar, preventing single-neuron recording. While we have largely solved the problem of mechanical or electrical failure of the electrode itself, much less is known about the long term immunological response to implantation of a microelectrode, its effect on neuronal recordings and, of greatest importance, how it can be reduced to allow long term single neuron recording. This article reviews materials approaches to resolving the glial scar to improve the longevity of recordings. The work to date suggests that approaches utilizing bioactive interventions that attempt to alter the glial response and attract neurons to the recording site are likely to be the most successful. Importantly, measures of the glial scar alone are not sufficient to assess the effect of interventions. It is imperative that recordings of single neurons accompany any study of glial activation because, at this time, we do not know the precise relationship between glial activation and loss of neuronal recordings. Moreover, new approaches to immobilize bioactive molecules on microelectrode surfaces while maintaining their functionality may open new avenues for very long term single neuron recording. Finally, it is important to have quantitative measures of glial upregulation and neuronal activity in order to assess the relationship between the two. These types of studies will help rationalize the study of interventions to improve the longevity of recordings from microelectrodes.

## 1. Introduction

In the past few decades, recording microelectrodes have been utilized to simultaneously acquire information from multiple, single neurons that encode for the intention to move in awake, freely moving animals. For example, the first demonstration of this type of brain-machine interface [1] used arrays of microwires to record multiple, single-neurons from the sensorimotor cortex of rats that encoded the intention of the animal to move its forelimb. The neuronal activity was combined into a population function that faithfully encoded the position of the limb and was used, in real-time, to control a robotic arm that provided a water reward to the rat. Importantly, this study demonstrated that, over time, the animal could learn to control the robotic arm in the absence of limb movement, suggesting that neuronal activity could be used to register the intention to move in patients who have lost control of their limb, for example after spinal cord injury [2] or who have lost a limb due to amputation. The implementation of recording microelectrodes in the human brain holds promise for other therapeutic treatments as well. Every year millions of patients lose neural function due to traumatic injury (i.e., brain injury or spinal cord injury), congenital conditions, degenerative diseases (e.g., amyotrophic lateral sclerosis) or other neural disorders [3]. While damage to the peripheral nervous system (PNS) can often be repaired, repair of the damages central nervous system (CNS) has been less successful, leaving patients with permanent disability. Therefore, microelectronic devices that tap into the system and repair or by-pass the injury or disease are needed. 

A good example of a successful neural implant that is in use today is the prosthetic cochlear implant used for the treatment of deafness. Within a normal ear, hair cells receive the auditory signal as a pressure wave, are deformed and convert the wave frequency into action potentials in the adjacent auditory neurons [4]. These action potentials are then transmitted to the CNS and ultimately perceived as sound. For people with impaired hearing, cochlear implants bypass the damaged hair cells and stimulate the auditory neurons directly, thereby completing the neural circuit to the CNS [4]. However, these implants, like other successful neural implants (e.g. stimulators for Parkinson’s disease) provide information to the nervous system via microstimulation. Much less success has been accomplished with implants designed to record information from the CNS.

The major problem with neural implants for recording single neurons, the focus of this review, is their failure to provide long-term *in-vivo* recordings. Two major reasons have been noted for the failure of such implants: (i) the mechanical failure of the implant itself, typically due to failure of the cable from the electrode to the signal conditioning device or loss of polymeric insulation of the microelectrode due to the corrosive extracellular environment and (ii) the formation of a glial scar surrounding the microelectrode rendering the implant useless because of its inability to record action potentials from single neurons [5]. While the former has been largely resolved in recent years, the latter continues to be a major impediment for the long-term *in-vivo* functionality of neural implants [6,7]. 

**Figure 1:**
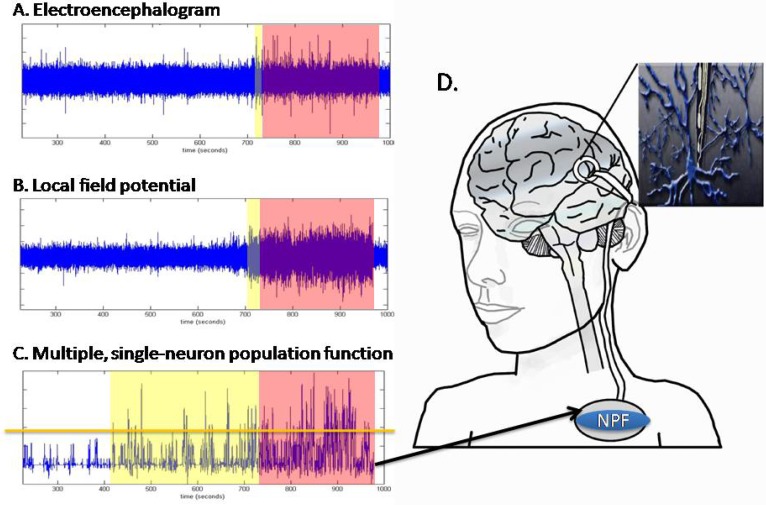
Representation of a brain-machine interface to identify changes in single-neuron activity early in the onset of a seizure. It is difficult to identify the activity in the EEG before the onset of behavior symptoms of a seizure (A). While local field potential can identify information earlier than EEG (B), changes in single neuron activity can identify the seizure onset minutes before behavioral symptoms are manifest (C). This could be used to control a brain-machine interface for epilepsy (D). A multisite, microelectrode, in this case our ceramic-based device, is shown implanted into the brain near neurons involved in the initiation of the seizures (D). An implanted control device would discriminate the single neurons from the analog signal, apply an algorithm (C) that is customized for the patient and report when a seizure was imminent or provide drug or charge delivery to prevent the onset of the full seizure. Data in A-C is from the rat after injection of kianic acid to induce seizures. Red areas indicate the behavioral manifestation of the seizure. Yellow areas indicate when the seizure could be detected by EEG (A), LFP (B) or multiple, single neuron activity (C). The orange line in C represents a threshold for the neuron population function (NPC). When the value of the NPC exceeds the threshold, a seizure is predicted.

Several different types of microelectrodes have been developed to record multiple, single-neurons simultaneously in awake, freely moving animals [8]. Recently, they have been used as an interface for a Brain-Machine Interface (BMI) that extracts command signals for the intention to move from the brain and uses the signal to move a cursor on a computer screen [2,9,10]. Moreover, it has recently been suggested that microelectrodes recording multiple, single-neurons could be used to identify the onset of seizure activity within neural circuits before the propagation of the seizure and, perhaps, in time to prevent further propagation [11] (Figure 1). However, *in-vivo* human applications involving the use of recording microelectrodes require the ability to chronically record action potentials from ensembles of single neurons indefinitely or at least for decades [5,12,13].

**Figure 2:**
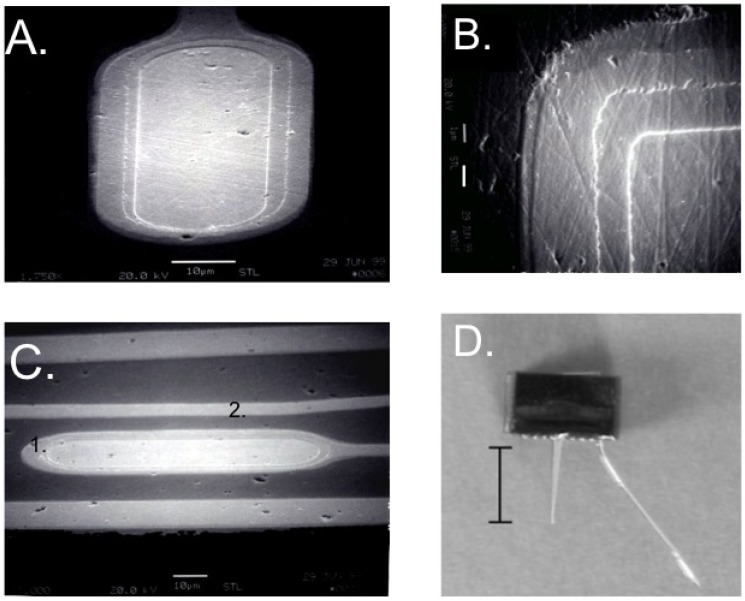
An important advancement in recent designs of successful microelectrodes is the insulating material. Thin film deposition of material that can insulate the conducting line of the microelectrode have improved the functioning of the microelectrode. Here we show an example of a ceramic-based microelectrode. A. The recording site is platinum (center) and layers of thin film ceramic are applied around the recording site and on the conducting lines using ion-beam assisted deposition. B. A close-up of the recording sites and the layers of insulation. C. A recording site and conducting line not yet removed from the ceramic substrate. C1 is a recording site. C2 is a conducting line from a downstream recording site that is completely covered in a thin film of ceramic. D. A completed microelectrode. The scale bar in D is 5 mm. Reformatted from Moxon, K.A.; Leiser, S.C.; Gerhardt, G.A.; Barbee, K.A.; Chapin, J.K. *IEEE Trans. Biomed. Eng.* **2004**, *51*, 647-656 [20]. © 2004 IEEE.

**Figure 3:**
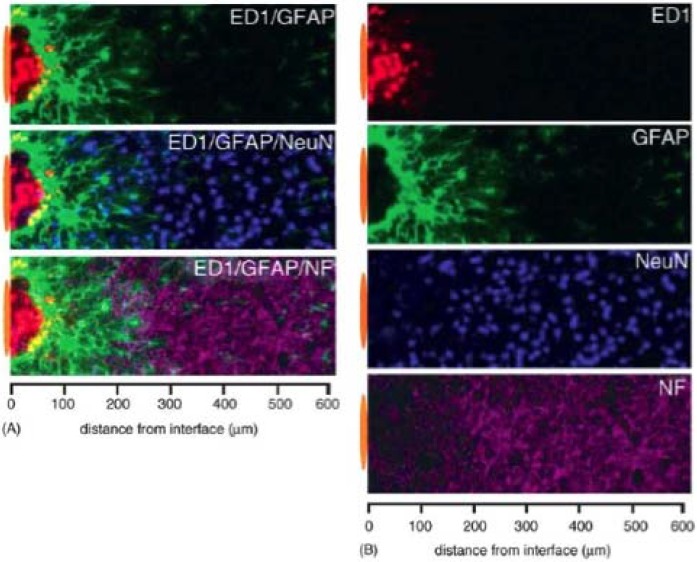
Stratification of cellular immunoreactivity using cell-type-specific markers at the microelectrode–brain tissue interface. Representative images collected from two adjacent sections of an animal with a 4-week microelectrode implant illustrate the general appearance of the foreign body response characterized by minimally overlapping inflammatory (ED-1) and astrocytic (GFAP) phenotypes adjacent to the implant interface. The area of inflammation and intense astrocyte reactivity contains a reduced number of NeuN+ neuronal bodies and a loss of neurofilament (NF) density. The position of the microelectrode is illustrated by the orange oval (drawn to scale) at the left of each image. Images were captured in grayscale and pseudocolored for illustration. Reprinted from Biran, R.; Martin, D.C.; Tresco, P.A. *Exp. Neurol.* **2005**, *195*, 115-126. Copyright (2005), with permission from Elsevier [21].

Unfortunately, long-term use of recording microelectrodes has not been realized. This failure is hypothesized to be due ***not*** to the electrical failure of the devices (Figure 2) but instead due to the biological response elicited by the insertion of the microelectrode (Figure 3). Current studies show loss of discriminable single unit action potentials within months of implantation [14,15,16,17] even when superior insulating materials are used such as ceramics (refer to Figure 2) or Parylene C [18,19]. For most microelectrode types, the loss of these recordings is not due to failure of the electrodes but rather due to reaction of the surrounding tissue that results in the formation of a glial scar and prevents the recording sites of microelectrodes from being close enough to single neurons to record their activity. 

Many studies have attempted to identify appropriate materials that would lessen the activation of glia after microelectrode insertion in order to improve the neuronal recordings. Studies include testing of different materials, assessing the effects of material properties including size, shape and surface texture and final the adding novel surface coatings. In this review, we will examine what is known about the reaction of the CNS to electrode insertion, what is important for long-term single neuron recording and what has been done to improve single neuron recording. There are clear principles emerging that are able to guide future studies into the development of new microelectrode devices. These principles include the following. First, it appears that a bio-active microelectrode that will interact appropriately with the CNS environment is needed. Second, it is becoming clear that rough surfaces, especially nanostructured are more conducive to healthy neurons than smooth surfaces, presumably because they mimic the extracellular matrix. Third, the biomechanical properties of the material are important but it is unlikely that any electrode device will be able to match the biomechanical properties of the brain. Therefore, un-tethering the microelectrode will be the best approach, and we present some recent, novel ideas to accomplish this.

## 2. The Brain’s Initial Reaction to the Trauma of Insertion

There is a significant body of work on the brain’s response to traumatic brain injury, and, recently, a better understanding of the immunological and cellular response to insertion of a microelectrode has been developed. However, it is still unclear how this response interferes with single neuron recording months later. The initial response is complex and involves many interrelated processes. Importantly, the long-term loss of neuronal recordings is the result of two events: the mechanical trauma to the tissue during insertion and the long-term response of the tissue to the foreign body [11]. Each event shares the same cell types but has different cellular pathways.

Mechanical trauma due to insertion of the microelectrode has been well studied. When inserted, the mciroelectrode tears through the tissue damaging neurons and glia, thus exposing the extracellular environment to intracellular proteins [22,23]. In addition, even if one is careful to avoid surface blood vessels, complete insertion of the microelectrode is likely to tear small capillaries, thus damaging the blood brain barrier (BBB) and exposing the extracellular environment of the brain to blood proteins [11,24]. The exposure of the extracellular environment to both intracellular and blood proteins initiates a cascade of events that can help to remove the damaged tissue and debris and heal the tissue or, if the damage is severe, create a glial scar that walls off the microelectrode from recording single neurons.

**Figure 4:**
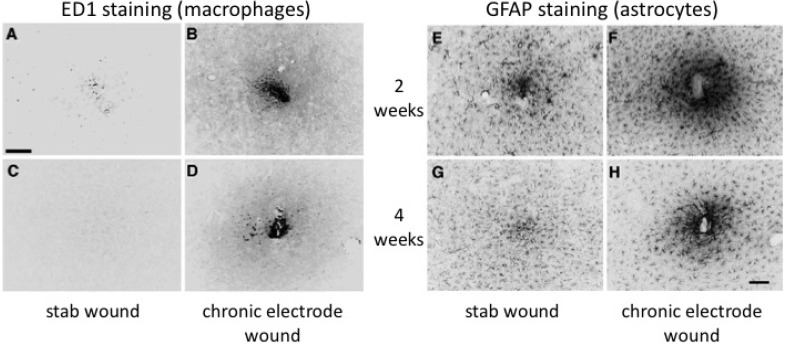
Figure ED1 and GFAP reactivity to stab wounds (A, C, E, G) and chronically implanted microelectrodes (B, D, F, H) at 2 (A– B, E– F) and 4 weeks p.i. (C–D,G– H), respectively. Representative fluorescence images demonstrate that ED1 and GFAP reactivity was more intense and extended to a greater distance around implanted microelectrodes (B, D, F, H) compared with time-matched stab wound controls (A, C, E, G). Scale bar = 100 microns. Reprinted from Biran, R.; Martin, D.C.; Tresco, P.A. *Exp. Neurol.* **2005**, *195*, 115-126. Copyright (2005), with permission from Elsevier [21].

The second major effect arises from the continued presence of the microelectrode in the neural tissue and is similar to the foreign-body response that has been well described for other tissues of the body [25]. If the brain is subject to a stab wound with a device about the size of a microelectrode within six months it will be difficult to identify the location of the wound if the procedure was completed under sterile conditions (Figure 4). In this instance, a stab wound was created by inserting the device, withdrawing it, then replacing the dura and skull over the stab site [21]. However, if the device used to create the stab wound is left in place, a glial scar can form around the device, effectively walling it off from the healthy neural tissue [26].Therefore, the initial damage done by microelectrode insertion, coupled with the continuous existence of the microelectrode, creates an environment that does not allow healthy neurons to remain close enough to the electrode to be recorded. 

To better understand the brain’s response to implanted microelectrodes, this section will briefly examine the different types of cells involved in the response and how this response is naturally regulated. Understanding the biological response is important for the development of bioactive materials that can help control the response and increase the longevity of single neuron recording. Next, the effect of this response on neurons around the microelectrode is discussed followed by ways to assess the effect.

### 2.1. The Cellular Response

A review of the cells involved in the response to microelectrode insertion is provided by [6]. Here, we restate some of the information from that review for context and extend it with additional notes. In the event of injury to cells and damage to the BBB, intracellular and blood proteins are released into the extracellular space producing a response by microglia, astrocytes, macrophages and leukocytes through a complex cascade of signaling molecules [5]. Each of these cellular responses plays an important function in protecting the brain from infectious disease and further injury [23,27,28,29]. The three best studied cell types directly involved in the brain’s response to injury are microglia, macrophages and astrocytes [30]. Macrophages can enter the brain when there is damage to the blood-brain barrier and become indistinguishable from activated microglia [24]. Together, microglia and astrocytes are known as glia and these cell types make up more than half the cells in the brain. For purposes of this review, when we refer to glia we include macrophages because after electrode insertion, the BBB is damaged and these cell types are, therefore, involved. In response to a traumatic injury to the brain, microglia are the first to be activated, usually within 24 hours of the time of injury, followed by the astrocytes within a week [31]. This activation includes physical changes to cell morphology including hypertrophy, expression of different surface proteins that act as signals to attract more glia to the site of injury and changes in the release of neurotrophic factors [32,33]. events can have both positive and negative effects on the outcome because the same mechanisms that can aid in the repair of neural tissue, can, if damage is severe, create a toxic state that further damages nearby, otherwise healthy, neurons [34].

Activated microglia initiate a complex chemical signaling cascade that increases the production of neurotrophic factors through regulation of cytokines and their influence on astrocytes [35,36,37,38,39]. The increase in neurotrophic factors may promote survival of neurons. However, if the injury is severe, high concentrations of neurotropic factors and negative effects of cytokines [24,37,40] can lead to further cell death [30,41,42]. In a similar manner, while recruiting glia to help repair the damage after electrode insertion is beneficial, the combination of hypertrophy and increased cell number can contribute to inflammation at the site of injury, which, if left unchecked, can damage neurons [43].

Macrophages similarly have beneficial effects that could be exploited, but over activation leads to negative effects on microelectrode recording capabilities [24]. Macrophages are not normally found in neural tissue but exist within the vascular system. When blood vessels within the brain are severed, monocytes from the blood are recruited into the neural tissue via the break in the BBB and are induced into morphological changes to become macrophages [24]. Similar to microglia, macrophages are responsible for dissolving cellular debris by secreting protolytic enzymes and removing the debris via phagocytotsis [42]. They also fuse together to form foreign body giant cells, which causes extreme inflammation. This excessive inflammation can lead to cavitations at the site of a gross injury [42]. Similar to the conclusion regarding microglia, since insertion of a microelectrode must damage the BBB, some proliferation of macrophages to clean up the damage is necessary. However, this proliferation must be managed by an active process that eventually down-regulates the response to allow the microelectrodes to remain in contact with healthy neurons for long-term, chronic recording. 

**Figure 5:**
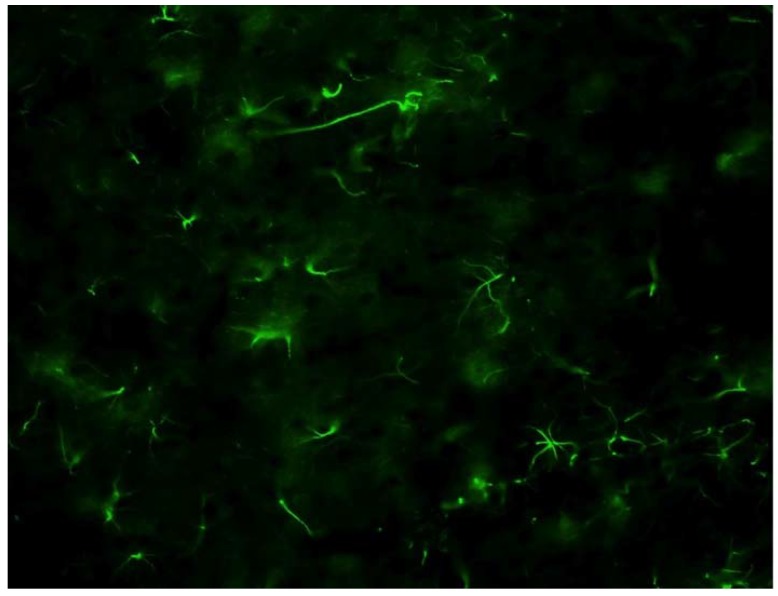
Resting astrocytes from healthy brain tissue stained with glial fibrillary acidic protein (GFAP). Astrocytes are named for their star like appears. They constitute 35-65% of brain cells and are critically important for normal functioning of neurons. Therefore, the goal is not to eliminate the glia but to regulate them with bioactive interventions.

The final important cell type in the brain’s response to electrode insertion is the astrocyte (Figure 5). Astrocytes provide mechanical support to neurons throughout their lifespan, provide growth cues to developing neurons and assist the transfer of nutrients across the BBB [35]. Therefore, it is important that any intervention not damage the astrocytic system. Astrocytes are activated by the presence of intracellular proteins in the extracellular space (i.e., if the cell’s membrane is damaged) and by signaling from activated microglia [44,45]. They are attracted to the site of injury, proliferate and become hypertrophied, all mechanisms that contribute to inflammation around the microelectrode [46,47]. Like microglia, they release neurotrophic factors [33], which is initially beneficial, but can contribute to overproduction creating a toxic environment. Astrocytes have been shown to create a physical barrier between healthy and damaged tissue which creates an inhibitory environment for neurite extension [31]. Therefore, similar to microglia and macrophages, some activation of glia is useful to support damaged cells, but, ultimately, the goal is to control this activation and have the tissue return to its normal, resting state following the insertion of the microelectrode. 

When considering the insertion of microelectrodes, the damage of insertion accompanied by the continued presence of the electrode shaft is likely to be sufficient enough to lead to excessive activation of glia. The optimal situation would be to allow for some activation of glia, sufficient to induce their beneficial effects, without overproduction, inflammation, and excessive damage. Unfortunately, little is known about where the effects of glia shift from being beneficial to detrimental and it is likely this threshold exhibits biological fluctuations depending on many variable biological factors. Therefore, these phenomena require more intensive study to be appropriately manipulated so that they can aid in the long term success of chronically implanted microelectrodes. However, given that some upregulation of glia are important to remove cells damaged from electrode insertion, it is likely that any intervention would have to be an active process, allowing for some upregulation followed by a signal to down-regulate the response and maintain healthy neuron-electrode interactions.

The conclusion that can be drawn from the information about these systems is that knowledge of the cellular response to electrode insertion does not immediately lead to a solution for successful implantation of the microelectrodes for chronically sustainable recordings. It is becoming increasingly evident that the goal is not to get rid of the glial response but to actively modulate it so that the beneficial effects of these cell types can continue to function effectively. The goal is to record single neurons. So the first question is how much glial scar is permissible without losing single neuron recordings. One important question, still unanswered, is whether the effects of electrode insertion outlined above impact the long term recording capabilities of a microelectrode despite the fact that the loss of recording occurs months after implantation and the brain recovers nicely from a simple stab wound. The different processes initiated during the acute phase of the response to electrode insertion (neuronal damage, damage to the blood-brain barrier, etc) combined with the continued existence of the microelectrode in the tissue may interact and initiate a cascade of events that may take months to progress to a state where single-neuron recording is lost. Traumatic brain injury research has shown that mechanical trauma can initiate progressive degeneration that continues long after the traumatic event [48]. Moreover, recent studies on the role of inflammatory responses around the microelectrode suggest that there is a continuous, local neurodegenerative state for at least 16 weeks after implantation [49]. However, the relationship between these processes and the loss of single neuron recordings is unknown.

### 2.2. Neuronal Cell Death and Migration 

Keeping in mind that the goal is to record single neurons regardless of the changes in glial regulation, two questions arise. First, how does the density of neurons around the microelectrode change with time? Second, (how) does the increased gliosis around the microelectrode contribute to the loss of neuronal recordings? As reviewed by [6], recent studies have suggested that the inability of the microelectrodes to provide long-term *in-vivo* recordings from neurons is due to the death of neurons immediately surrounding the electrodes, migration of neurons away from the microelectrode or a combination of the two. Therefore promoting neuron survival and a healthy environment for the neurons following electrode implantation will be important in ensuring single neuron recording capability. 

While some of the factors that contribute to the glial scar (size of the device inserted, speed of insertion, etc) have been well studied and are reviewed next, the relationship between single neuron recording and glial scar formation is less well understood despite the fact that considerable work has been done understand the time course of the cellular up-regulation following microelectrode insertion [15,21,50]. Therefore, in any study of chronic electrode function, it is imperative to record single neurons activity so that this relationship can be clarified. However, this is rarely done. In fact, most studies do not even use functional microelectrodes. The likely reason for this is that the studies to date have only looked at changes in gliosis within weeks of implantation when most neuronal recordings are stable. However, if there are significant changes in gliosis, it would be interesting to know if there was any impact on the neuronal recording.

Neuronal death following chronic electrode implantation occurs in two ways. First when the microelectrode is inserted into the cortex it breaks and tears neurons which can lead to cell death. Second, the accompanying glial response could further damage neurons due to the persistent existence of the microelectrode. In support of this, [21], observed a strong inverse correlation between changes in the density of neurons and the density of microglia around the microelectrode. In fact, they and others [15,50] found activated microglia tightly packed near the microelectrode and on the microelectrode that was removed from the tissue. Biran *et al*. [21] concluded that either (i) microglia activation leads to neuronal loss or (ii) neuronal damage at the site of injury leads to excessive glial activation due to another mechanism or (iii) neurons are displaced from the electrode site by the recruitment of glia to the site of injury. One possible scenario cited by [21] for the loss of neurons around the microelectrode is that the glial activation leads to a phenomenon called “frustrated phagocytosis”. The phenomenon is tagged as a positive feedback mechanism where the activated microglia continuously produce cytokines due to their inability to clear the insoluble microelectrode. The death of neurons is therefore due to the toxicity created by cytokines such as TNF-α, IL-1β and prostaglandins [21]. This is in keeping with the time course of neuron recording loss and the fact that there is great variability in the timing of the loss of neurons.

A previous study conducted by [42] provides further support to this theory. Both studies are in agreement that the death of neurons is due to cytokine toxicity; however [42] suggests that some degree of mechanical damage also occurs to neurons due to astrocyte migration. Astrocytes are the primary support cells for neurons in the CNS [51] and migrate away from the macrophage rich zones. Therefore mechanical damage to the neurons occurs as they were stretched, moved, pulled and torn between the migrating astrocytes [42].

### 2.3. Evaluating the Success of Response Reduction

The ability to understand the brain’s response to electrode insertion on the cellular level is important for directing interventions to optimize the ability to obtain neuronal recordings indefinitely. Because the cells involved in the reaction to the inserted electrode (microglia, astrocytes and macrophages) express novel proteins on their surface when they are in their activated state, immunohistochemistry is generally used to label cells that express these proteins and the amount of labeled tissue can be quantified. Therefore, utilizing immunohistochemistry to classify and quantify the cellular response is an integral part of evaluating response reduction. 

Proteins that are specific to the previously discussed cells of interested have long been identified and studied in the literature [52]. The most common labels are glial fibrillary acidic protein (GFAP) for reactive astrocytes, ED-1 for reactive microglia, and staining for neuronal nuclei, NeuN. During conventional immunohistochemical analysis, these proteins are associated with a primary antibody that will bind to the protein of interest. Once the primary antibody has bound to the protein, a second antibody tagged with a detection agent is then added to bind to the primary antibody. This allows for visualization under a microscope for further analysis [52].

**Figure 6:**
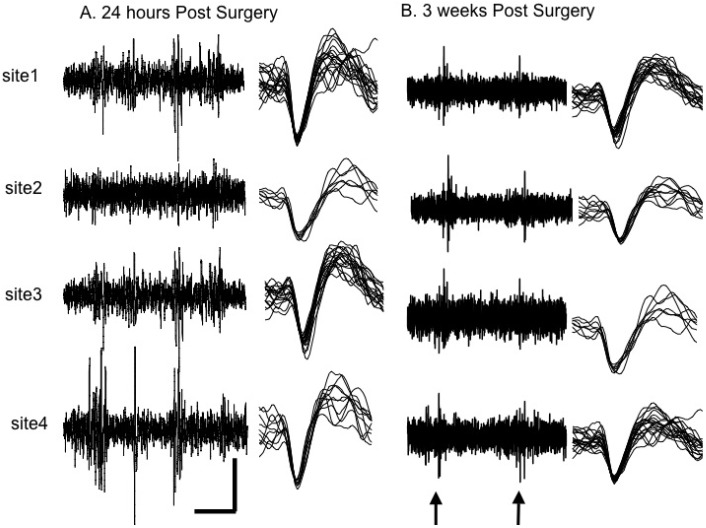
Comparison of chronic recordings of multiple, single-neuron activity from a lightly anesthetized rat at (A) 24 hours post-surgery and (B) three weeks post-surgery. (A) At 24 hours post-surgery, the recording trace shows that multiple single neurons were recorded on each of the four recording sites. Example of a single neuron action potential discriminated from each recording site is presented next to the recording trace. (B) Three weeks post surgery, activity dependent action potentials were recorded during deflecting of a single whisker (in this case whisker E4). Arrows at the bottom of the analog trace show times of whisker deflection. All four sites responded to the deflection of the same whisker suggesting that all four recording sites of the CBMSE array were implanted into the same cortical barrel. Representative single neurons for each channel were discriminated and are displayed adjacent to the trace recording from each of the four recording sites. Horizontal scale bar 50 msec. Vertical scale bars 100 mV. Reproduced from Moxon, K.A.; Leiser, S.C.; Gerhardt, G.A.; Barbee, K.A.; Chapin, J.K. *IEEE Trans. Biomed. Eng.* **2004**, *51*, 647-656 [20]. © 2004 IEEE.

The most important practical indicator of successful reduction of the cellular response to microelectrode insertion is the ability to continue to obtain consistent recordings of single neuron action potentials for years. Moreover, since the response of glia to microelectrode implantation is highly variable, to assess the effect of gliosis on neuronal recording, the activity of single neurons must be assessed. Studies that also assess the density of neurons around the microelectrode are useful. However, most recording sites can discriminate only 1-3 neurons successfully and the relationship between neuron density and the ability to discriminate single neurons is unknown. These single action potentials are the signal of interest and therefore, as the ability to record these single action potentials decreases, so does the ability to use the information for different applications. Therefore, when studying different materials for microelectrodes, it is important assess the effect on single neuron activity (Figure 6) as well as glial scar formation. 

Several groups have achieved recordings from current implantation techniques that last about a year. For example, [53] were able to record discriminable single-neuron action potentials from the brain of a monkey for more than a year. Importantly, they quantified their recording stability through four measures: “spike shape, spike train autocorrelograms, spike frequency, and range of peak amplitudes.” Spike shape was evaluated by a vector made from three spike characteristics. The first characteristic was the amplitude of the first phase of the spike, the second characteristic was the amplitude ratio comparing the first and second phases of the spike, and the third characteristic was “inter-peak-interval” of the spike. These three components yielded a numerical vector that could be normalized and used to compare the spike shape between different recording sessions. These types of quantitative measures are useful for comparing results across groups. 

Other groups have quantified the effect of gliosis on the impedance at the recording site—tissue interface [54]. It has been shown that the increase in glia around the microelectrode increases the impedance at the electrode-tissue interface [54,55]. The concept underlying this measurement is that as the glial scar builds up, the buildup increases the impedance between the recording site and the tissue. One possibility then is that impedance would be inversely correlated with the quality of the recording. *In vitro*, [54] demonstrated that cellular adhesion to the recording site surface yielded changes in the impedance varying from 20% to 80% and this change was shown to persist for a period of weeks. In agreement, both [49,55] showed that the impedance changes were correlated to GFAP expression. However, no relationship between changes in impedance and single neuron recording *in-vivo* were found [20,54] Therefore, it is not clear that this change in impedance is related to the loss of neuronal recordings. 

Another method to assess the effects of microelectrode insertion on the surrounding tissue was to measure changes in pH [56]. Interestingly, they found more coagulated blood along tracks where prolonged acidosis occurred, suggesting that the variability in pH could be related to damage to the BBB. Therefore, measures of pH could be another method to assess the effects of interventions to mitigate the response to microelectrode implantation.

Finally, techniques are improving for assessing the release of signaling molecules in response to the injury created by microelectrode implantation. These signaling molecules or cytokines are proteins and therefore changes in their gene expression can be assessed to better understand the effects different interventions on the glial response. For example, Bellamkonda and colleagues have used reverse transcription quantitative polymerase chain reaction (RT-qPCR) both *in vitro* [57] and *in vivo* using in situ hybridization [58] to assess changes in cytokine concentration. Since the loss of neuronal recordings occurs months after the gliosis appears to stabilize around the microelectrodes, this approach could be used to identify changes that are occurring at the time that the neuronal signals are lost.

### 2.4. Effect of Initial Microelectrode Insertion on the Glia Response

Several investigators have studied the effects of different techniques for microelectrode insertion into the brain to develop methods to minimize negative effects on the tissue from insertion. The methods utilized to attempt to minimize deterioration of the tissue around the inserted microelectrode include improving surgical techniques, modifying the speed of microelectrode insertion, and optimizing the geometry of the microelectrode. Each of these approaches is examined next.

After the skull is removed, the dura mater, a thick connective tissue layer, is encountered which is easily removed prior to electrode insertion. The next layer, the thinner pia mater, is very close to surface blood vessels and the brain. It is more difficult to remove and often left intact when inserting a microelectrode. Unfortunately, this pial surface makes it more difficult to insert the microelectrode and extensive dimpling of the surface can occur before the pia is broken and the microelectrode enters the brain. Of course, the speed and shape of the electrode tip influence the ability of the microelectrode to push through the pia [59] In fact, [59] found that blunt tips inserted at slow speeds created severe compression or dimpling of the brain when the pia fails to break and also produced more displacement of microvasculature. This often resulted in rupture of the vessels. These studies were confirmed by studies measuring pH shifts immediately after electrode insertion at different speeds through the pia [56]. Therefore, the pia should be removed, either surgically or with a collagenase [60,61] before the microelectrode is inserted. However, if the pia is to remain intact, sharp microelectrodes, inserted rapidly have been used to yield good recordings of single neurons for months [62].

**Figure 7:**
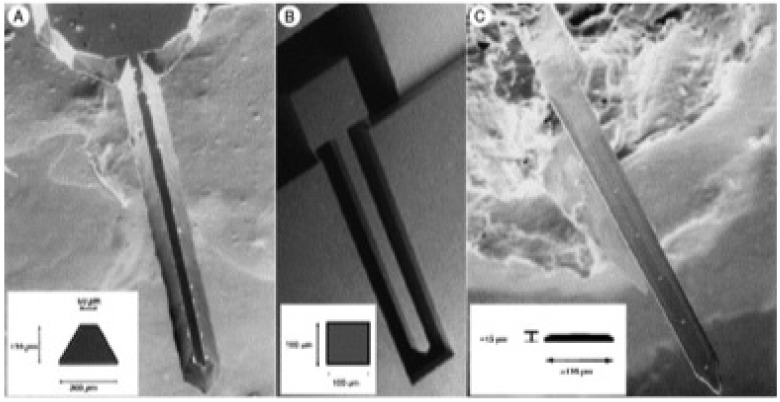
The effect of different shapes and sizes of microelectrodes have been tested. While there appeared to be effects at short time windows of the different devices, the tissue response to the implanted devices was basically identical at 6 weeks. The devices were not functional so no single neuron activity could be recorded. Reprinted from Szarowski, D.H.; Andersen, M.D.; Retterer, S.; Spence, A.J.; Isaacson, M.; Craighead, H.G.; Turner, J.N.; Shain, W. Brain responses to micro-machined silicon devices. *Brain Res* **2003**, *983*, 23-35, Copyright (2003), with permission from Elsevier [15].

Investigators have explored the influence of microelectrode shapes, especially at the tip of the microelectrode on the brain’s reaction to microelectrode insertion. However, well-controlled studies were not performed until the last half decade. Szarowski *et al*. [15] performed a comprehensive study on the long-term success of a variety of surface modifications and their effect on the chronic tissue reaction to the microelectrode (Figure 7). They studied size, surface texture, cross sectional shape, tip geometry, and insertion technique to determine the effects of these aspects on the short and long term reaction to microelectrode insertion. Microelectrodes with either a 2,500 µm^2^ (50 × 50), 10,000 µm^2^ (100 × 100), or 16,900 µm^2^ (130 × 130) cross sectional area and either trapezoidal, square, or ellipsoidal cross sectional geometries were compared against one another. Smooth surface textures or micrometer rough surface textures, blade or rounded tip geometries, and slow and fast insertion techniques were also compared. Using qualitative estimates of the amount of GFAP and ED-1 staining, they concluded that the size of the microelectrode had an effect on cellular upregulation one-week after insertion, but this effect was not evident after six weeks. In another study [63], differences in GFAP expression in response to implants with a diameter of 12 microns was quantitatively assessed and compared to an implant of 25 microns. They found no effect at two weeks but significantly less GFAP expression around the 12 micron diameter implant compared to the 25 micron implant at four weeks. The lack of an effect at two weeks might have been due to the need to guide the 12 micron implant during insertion. While longer term studies need to be performed, these results suggest that quantitative measures are important and that size may influence glial response. 

## 3. Bioactive Interventions to Modulate Gliosis

Effective recording of action potentials from single neurons is possible only when the distance between the electrode and nearby neuronal cell body is small. Of course, the size of cell influences the size of the action potential and, therefore, the ability to record the cell. Large pyramidal cells have been recorded during acute recordings (same day) from high impedance microelectrodes array as far as 300 microns [64,65] and at least 100 micron [66]. One study using simultaneous intracellular and extracellular recordings suggested that in the hippocampus, the cell must be within 140 microns of the recording site in order to see any extracellular activity. In practice, recordings from chronically implanted microelectrodes are likely made from neurons within 100 µm of the recording site in order to reliably discriminate the action potential from background activity. The invasion of activated microglia and macrophages and the loss of healthy astrocytes and neurons near the microelectrode can cause single neuron recordings to be lost within a year of implantation. Therefore, several investigators have tested the use of bioactive interventions to maintain closer contact of the recording site to the neurons of interest.

### 3.1. Increasing Adhesion between the Microelectrode and the Neural Tissue

One approach explored to improve the recording capabilities of microelectrodes was increasing the adhesion between the recording site and the neural tissue. The idea behind this approach is that micromotion between the brain and the device produces chronic strain in the tissue which is at least partially responsible for the sustained tissue response to the device [67]. Modeling the impact of micromotion suggested that the degree of adhesion affects the strain distribution in the vicinity of the electrode [68,69]. However, things are likely to be more complicated *in-vivo*. For example, the adhesion changes not only the distribution of strain but the type (i.e., shear vs. normal) as well. Furthermore, little is known about the response of brain tissue to these levels of strain over long time periods.

However, recent studies have tried to address these issues *in-vivo*. For example, Bellamkonda and colleagues developed coating for microelectrodes to encourage integration of neural processes into the coating. They used a thin coating of polyethyleneimine (PEI)-laminin applied using their layer-by-layer (LbL) approach [57]. Using silicon wafers as the substrate, the authors created multilayers of oppositely charged PEI and laminin. Thickness measurements of the manufactured films showed that even coatings of up to 11 nm could be manufactured using the LbL technique [70]. To check the biocompatibility of the films produced, the authors performed two *in-vitro* assays, a neuron cell adhesion assay and a neurite outgrowth assay. The results from both assays showed that PEI-LN coatings were highly biocompatible [70].

Having obtained positive results from the *in-vitro* studies, the authors carried out *in-vivo* studies in rats [57] to assess longer term effects of cell adhesion. PEI-LN coated electrodes were implanted into the cortex of male Sprague-Dawley rats. As controls they implanted uncoated electrodes into the cortex of the same animals. By four weeks post implant the coated microelectrodes had less ED-1 staining and less GFAP intensity of staining around the microelectrodes with no effect on the distribution of neurons. Interestingly, they did find significantly greater increases in cytokine release around the coated microelectrodes within 24 hours of implant compared to the uncoated microelectrodes using realt-time PCR. These data suggest that the coating may produce an enhanced initial reaction to the damage caused by insertion that produces an enhanced ‘clean-up’ of the immediate effects of insertion resulting in less gliosis at four weeks [57]. These results suggest that there is an important relationship between the effects of the immediate damage due to microelectrode insertion and the chronic response to microelectrode insertion. 

While the immediate impact of electrode insertion likely has consequences for the long-term glial response, improving cell adhesion to the microelectrode is not a straight-forward approach. Recent work examining the extraction force necessary to remove a microelectrode from the brain (presumably a measure of tissue integration) documented that the intensity of GFAP staining around the microelectrode was well correlated to extraction force, better than staining of any other cell type including neurons [71]. This result suggests that it is the upregulated astrocytes that are mainly responsible for the tissue adhesion. Therefore, significant work needs to be done to understand the best approach to creating and maintaining electrode-tissue integration. One possibility is to carefully select the types of proteins used to attract tissue to the microelectrode and ensure that neurons, perhaps as well as glia, are attracted [72].

### 3.2. Polymer Coatings for Microelectrodes 

There are generally three approaches to using bioactive molecules to improve microelectrode recording: delivering drugs via microfluidcs, systemic injection of drugs to minimize the immunological response, and attaching biomolecules to the surface through some type of chemical bond (conjugation). Some investigators have proposed to incorporate microfluidics to deliver novel drugs to the insertion site in order to attenuate the biological response to microelectrode insertion [73]. In order to make these fluid channels beneficial though, fabrication technique must be utilized that can incorporate these channels without greatly increasing the size of the microelectrode tip. Drug delivery channels have been incorporated into the microelectrode tips and used to deliver small amounts of drug [74,75] via passive diffusion. On problem is that the channels can get clogged so that passive release is unreliable. Moreover, the diffusional distance of the drugs is limited. Retter *et al.* [76] used pressure mediated release and found that it was possible to quantitatively control the volume of drug delivered. However, as yet, these microfluidic device have not been used in chronically implanted animals so their long-term viability is unknown. 

**Figure 8:**
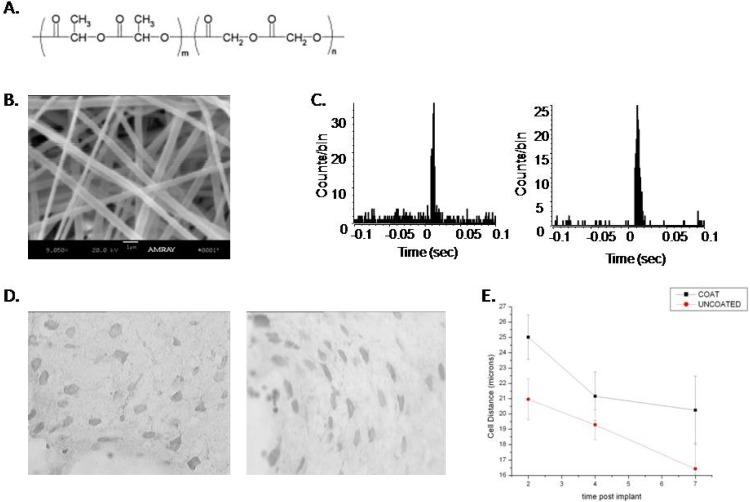
A. Structure of poly(lactide-co-glycolide) (PLAGA). B. Scanning electron micrograph of the size and shape of the fibers (scale bar 1 micron). Fibers were electrospun using 20% PLAGA solution with a 16 gauge needle onto ceramic-based microelectrodes creating a layer 5 microns thick. C. Comparison of perievent histograms recorded from microeletrodes with and without a coating of PLAGA fibers four weeks post-implantation into the barrel field cortex of the rat. Single cells could consistently be recorded from both coated and uncoated microelectrodes (n = 6 electrodes per group) and cells responded to whisker deflection. D. Animals were sacrificed at 2, 4 and 6 weeks post implant and the tissue was stained for neurons using NeuN. E. The distance of cells from the neural interface were measured and compared between electrodes that were coated with PLAGA and those that were not. Cells found closer to the recording sites at 6 weeks compared to 2 weeks, presumably due to the subsiding of the initial inflammation in response to electrode insertion. However, cells around the coated microelectrode remained 5 microns further away than the uncoated microelectrodes for the entire 6-week period.

Another approach to drug delivery was to coat the microelectrode with biocompatible polymers that could then be loaded with drugs that are released and help to ameliorate the glial response. Willerth *et al.* [77] provide an interesting review of this approach for tissue engineering in general. Several studies have investigated this approach for microelectrode implantation. For example, poly (lactic-co-glycolic acid) (PLAGA) coated microelectrodes were manufactured to test their effect on gliosis and single neuron recording. PLGA, a biocompatible and FDA (Food and Drug Administration) approved polymer was electrospun into a 5 micron thick fiber mesh or coating around the ceramic microelectrodes that could serve as a scaffold for later drug delivery (Figure 8). 

For the *in-vivo* study, ceramic-based microelectrodes coated with the 5 micron layer of PLAGA fibers were implanted into the barrel field cortex of rats (Figure 8). Uncoated controls implanted into the opposite cortex of the same animals were used for comparison. Single neuron action potentials were recorded in response to stimulation of the whiskers at one week post implantation to ensure that the coating did not interfere with the ability to record single neurons (Figure 8C). Immunohistochemistry was then performed to look for the presence of astrocytes and neurons surrounding the electrode implantation site. The size of the glial scar and the density of GFAP positive astrocytes were not significantly different between the coated and uncoated microelectrode. Furthermore, there was no significant difference between the mean distance of neurons from the edge of the tissue. However, neurons around the coated microelectrode were consistently almost 5 microns farther away than for the uncoated, the same distance as the PLAGA layer (Figure 8E). These results suggest that when considering coating micorelectrodes to make them more bioactive, the thickness of the coating must be considered because it can increases the distance of the recording site from the neurons [78]. 

Recently, polyethylene glycol (PEG) was used to immobilize L1, a neural adhesion molecule, and tested to evaluate the ability to attach neurons [79]. L1-PEG was found to be as effective as laminin in neuron attachment and to increase neurite growth compared to laminin. Importantly, L1-PEG has significantly less glial adhesion, approximately 8 times less. The results suggest that the adhesion chemistry can be engineered to promote cell-specific responses. 

Another approach to overcome the effect that coatings around microelectrodes can have on increasing the distance between recording sites and neurons is to incorporate conducting polymers [80]. The use of these polymers was recently reviewed by [81] and will be only briefly discussed here. The most widely used conducting polymer for neural recording, polypyrrole, was originally developed as a biocompatible interface for stimulating cells [82,83] and neurons [51] in culture using methods of electrochemical polymerization. Recently, polypyrrole has been combined with bioactive molecules onto the surface of microelectrodes to improve the signal conduction at the recording site surface and to attract neurons [80,84,85]. These devices were tested both acutely and chronically [86]. These experiments were able to show high levels of function as well as preferential neuronal growth on the surface. Chronic testing showed that this approach could produce stable neural recordings at some sites after one week, following which time the recordings were lost [87]. A similar conducting polymer poly(3,4-ethylenedioxythiophene – PEDOT) was used to attenuate the loss of recordings encountered when microelectrodes were coated in a hydrogel [88]. Since conducting polymers are produced by doping a base polymer, their conductance is not as good as metals and the dopant can be lost. To overcome this limitation, carbon nanotubes were incorporated into polypyrrole films and shown to improve the electrochemical stability of the resulting conducting polymer in acute studies [89]. While, conducting polymers may provide improved interfaces between microelectrodes and neural tissue, more work is required to better understand this interface and how conducting polymers can improve neuronal recordings for very long term use. 

### 3.3. Enhancing Microelectrodes with Bioactive Molecules

Several different kinds of bioactive molecules have been added to microelectrodes to minimize gliosis in the hope of improving long-term neural recordings. Koyama *et al*. [41] investigated the effects of BQ788 on reactive astrocytes. BQ788 is an endothelin ETB receptor antagonist. Endothelin has been shown to regulate the function of astrocytes. Reactive astrocytes and activated microglia are the main cells that express the ETB receptor. Injury was induced in male rats by a unilateral stab wound using a razor blade. BQ788 was administered by continuous infusion and caused a significant decrease in the GFAP staining at two weeks post injury. However, this ETB receptor antagonist also produced an increase in the number of microglia. Therefore, endothelin has shown potential to be used in future experiments, though one must investigate the effects of this receptor antagonist for a longer time period. 

In a similar manner, [90] investigated the effect of anti-coagulation factor protein S (PS) on activated astrocytes. PS is a plasma protein expressed in cultured glial cells. Injury was induced by scratching cultured rat astrocytes *in vitro*. PS activity was measured by using a functional clotting assay in the presence of serum and in the absence of serum. At a concentration of 100 nM, PS suppressed the proliferation of reactive astrocytes by 50%. At a concentration of 300 nM it suppressed the proliferation by 90%. The mRNA expression of PS was also investigated, and a marked increase in expression was observed 15 hours after injury. This up-regulation of PS mRNA expression after injury suggests that PS may be involved in regulation astrocyte proliferation after injury.

In another set of experiments, tumor necrosis factor-β1 (TNF-β1) was administered to cultured astrocytes, since TNF-β1 is known to be a strong inhibitor of astrocyte proliferation [37]. Cell growth was assessed by incorporating [3H] thymidine and bromodeoxyuridine BrdU in cultured astrocytes for 24 hours. Reactive astrocytes were reduced to 50% of control level when TNF-β1 was administered at a concentration of 20 ng/mL. Transforming growth factor-β1 (TGF-β1) is also known to inhibit proliferation of astrocytes [91] and was tested as a bioactive agent for microelectrodes by immobilization on dextran surfaces [92]. *In vitro*, proliferation of astrocytes was reduced by 57% suggesting that it too could be used to control gliosis after microelectrode implantation. 

Finally, it has been shown that electrodes produced with neurotrophic factors can reliably record single neurons from human patients and other primates and used to allow patients to control a cursor [10,93,94,95]. These electrodes are relatively large and have one recording site at the tip, making the recording of large numbers of single neurons, as would be necessary for restoration of movement, difficult. To overcome this limitation, hydrogels have been employed as a drug delivery mechanism from multisite microelectrodes to deliver BDNF [96] and release of BDNF was shown *in-vitro*. In addition, neural stem cells to release trophic factors from hydrogels on microelectrodes have been tested in an attempt to increase the longevity of single neuron recording. When the device was tested *in-vivo*, extensive gliosis at six weeks post implant occurred, likely due to breakdown of the hydrogel and the inflammatory response to the foreign cells in the hydrogel. 

In addition to modulating the glial response directly, anti-inflammatory agents have also been used to modulate receptors involved in the inflammation process [97]. Dexamethasone, an anti- inflammatory synthetic glucocorticoid, was administered to animals after surgical implant of microelectrodes into the brain. When systemically injected, the response of astrocytes was attenuated, but the microglia response was increased [98]. A similar effect of dexamethasone, slowly released from nitrocellulose coatings around microelectrodes, was found for astrocytes at four weeks after implant [99]. Local delivery of dexamethasone, by slow release from poly (ethyl-vinyl) acetate, was compared to systemic injection and found to be as effective at attenuating astrocyte proliferation but without the side effects of systemic injection [96]. In an effort to increase the duration of drug delivery, PLGA nanoparticles were loaded with dexamethasone and shown to release drug for more than two weeks [100]. As a deterrent to astrocyte proliferation at the microelectrode, dexamethasone is a promising bioactive molecule. 

Another anti-inflammatory agent, alpha-melanocyte stimulating hormone (alpha-MSH), was tested for use around microelectrdoes. Alpha-MSH inhibits cytokines involved in the inflammatory process, so it is predominately altering the effects of microglia. Using the nitrocellulose coating, alpha-MSH could be released for over two weeks and was shown to be active by inhibiting nitric oxide production from microglia *in vitro* [101]. However, each of these drug delivery approaches suffer from limited duration of release. To overcome this, alpha-MSH was immobilized on microelectrodes [58] and shown to be bioactive by reducing the release of nictric oxide *in vitro*. Further tests *in vivo* showed that ED-1 staining around the microelectrode was attenuated at four weeks post implant. If immobilized anti-inflammatory agents can be shown to be continuously bioactive, reducing the inflammatory response, it would be important to show that this can lead to longer neuronal recordings. 

**Figure 9:**
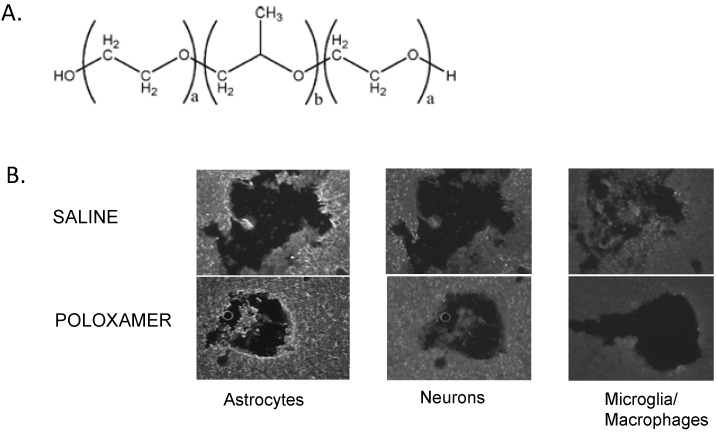
Effect of P188 on the reaction of tissue around the microelectrode. A. The structure of P188 a co-block polymer. B. Tissue was stained for astrocytes, first column (GFAP), neruons, second column (NeuN) or microglia/macrophages, third column (ED-1). All tissue is from the same animal. For the top row, tissue is taken from around an uncoated implanted microelectrode (saline). For the bottom row, tissue is taken from around a microeletrode coated with P188. The P188 coated microelectrodes show glial processes that are upregulated in a smaller band around the electrode and these glia are less hypertrophied. Moreover, the P188 coated microelectrodes show more neurons closer to the electrode and the density of neurons is greater. Finally, the P188 coated microelectrodes show decrease in microglia and macrophages around the electrode.

Neuroprotective agents are another type of bioactive approach to reducing the glial response to microelectrode insertion. One such agent that has received recent attention is poloxamer (P188). Poloxamer is a water soluble, amphiphilic, triblock copolymer that has been approved by the FDA to be used a skin cleanser. The polymer has two hydrophilic polyethylene oxide residues (PEO) on either side and a polypropylene oxide (PPO) residue in the center (Figure 9). 

Poloxamer, when added to mechanically injured cells, has been found to seal plasma membranes, promoting cell viability and hence subsequent recovery [102]. Due to its surfactant properties, it is believed to work by sealing damaged membranes, thus preventing contamination of the extracellular space by intracellular proteins and promoting cell survival [103]. By treating mechanically injured PC2 cells, a subline of rat pheochromocytoma cell line, with P188 *in-vitro*, the study showed that P188 promoted neuronal cell viability in a dose dependent manner [102]. We hypothesize that poloxamer could enhance neuron survival following electrode injury aiding in the repair of neurons damaged during electrode insertion. Preliminary studies suggest that Poloxamer can reduce the activated microglia around the electrode and increase the density of neurons (Figure 9).

## 4. Altering the Physical Structure of the Microelectrodes 

### 4.1. Modifying Surface Structure

Finally, recent *in vitro* studies are beginning to suggest that changes in surface structure on the nano scale level can have an important effect on neurons and glia. In fact, it has been shown that cell adhesion is higher for surface features in the ten’s of nanometer range compared to the hundred’s of nanometer range [104,105] studied the effect of carbon nanofiber coatings on astrocyte proliferation *in-vitro*. These fibers were either 60 or 200 nm and with either high or low surface energy. These results showed that microelectrode tips coated with fibers of smaller diameter and higher surface energy lead to a decrease in astrocytic adhesion. Since these cells are one of the primary cells believed to be involved in the encapsulation and isolation of the microelectrode *in vivo*, the group concluded that it may be possible to decrease the glial encapsulation of the microelectrode by manipulating the surface structure of the microelectrodes at the nano-scale [105]. 

**Figure 10:**
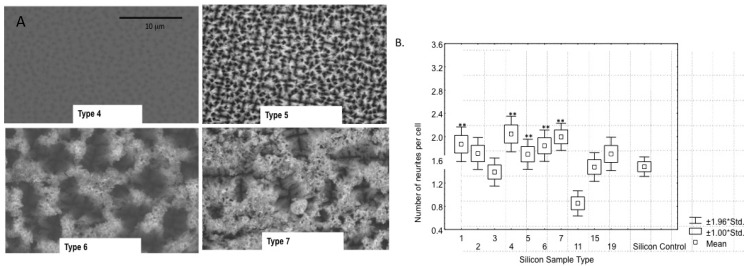
A. Etch parameters were varied and produced a range of porous silicon surfaces SEM was used to examine the porous structure. Sample 4 appeared slightly porous with very shallow, evenly-spaced, micrometer-diameter pores. The remaining three samples, 5, 6 and 7, were sufficiently porous. This trio was fabricated from n-type Si wafers doped with phosphorous. All photomicrographs were taken at 3,500x and the scale bar, 10 mm, is the same for all. B. Effect of etch parameters on bioactive properties *in vitro*. Samples were tested *in vitro* and the average number of neurites produced on PC12 cells after three day incubation were compared. Samples 1 and 4-7 produced significant neurite outgrowth from PC12 cells *in vitro*. Samples 4-7 were confirmed to be nanostructured surfaces based on their brilliant red glow under UV light compared to the other samples. ** represent significant difference compared to smooth silicon control [106].

**Figure 11:**
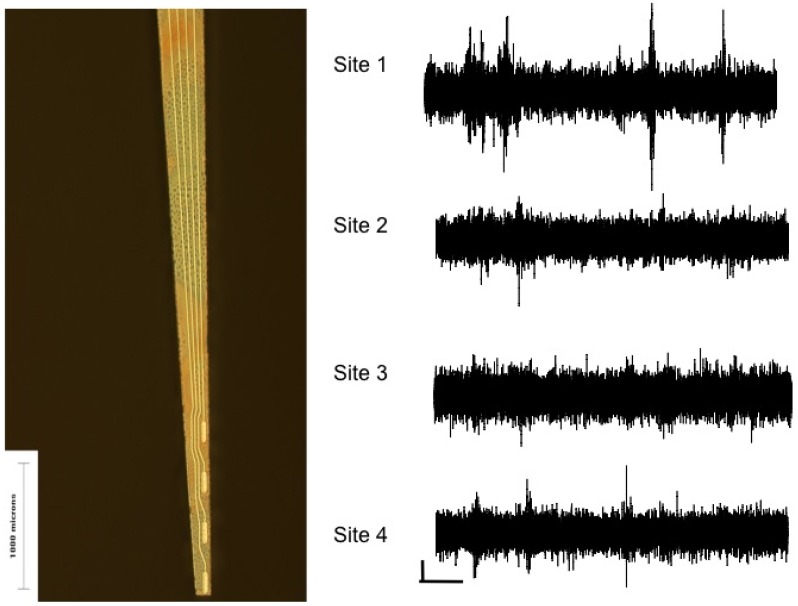
Thin film nanostrucutred porous silicon microelectrodes. A. Microelectrodes insulted with thin film ceramic could be produced from from bulk silicon wafers etched to create porous silicon surfaces. B. Single neurons could be recorded from the four recording sites patterned at the tip of the device. The electrode was implanted into the barrel field cortex, and neurons responded to whisker deflection as measured from the peri-stimulus time histogram (data not shown). Reformatted from Moxon, K.A.; Hallman, S.; Aslani, A.; Kalkhoran, N.M.; Lelkes, P.I. Bioactive properties of nanostructured porous silicon for enhancing electrode to neuron interfaces. *J. Biomater. Sci. Polym. Ed.* **2007**, *18*, 1263-1281 [107]. Copyright 2007.

**Figure 12:**
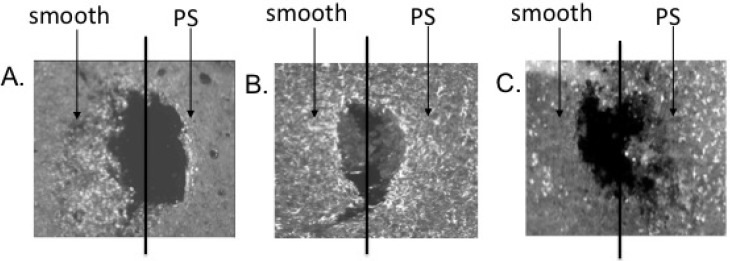
Immunohistochemical staining around PS-devices, where one side (left on each panel) is smooth and the other (right) is nanostructured porous silicon (nPS). A. GFAP expression, which marks astrocytes, shows less staining on the nPS side than the smooth side. Astrocyte activation around each electrode hole was quantified by measuring the fluorescence intensity within 50 micron increments from the edge of the hole left by the electrode. While the fluorescence intensity adjacent to the porous silicon surface was consistently less than that around the smooth surface, the differences were not significantly different at any distance. B. ED1, which stains for macrophages and microglia, is significantly reduced on the porous side of the electrode compared to the smooth side. Microglia and macrophage activation around each electrode hole was significantly greater adjacent to the porous silicon surface than the smooth surface. C. NeuN staining, which marks neurons, shows significantly more staining on the nPS side than the smooth side. This particular animal was sacrificed one week after the PS-device was implant. The black line was added to show how the smooth side was discriminated from the porous silicon. Neurons were more likely adjacent to the porous silicon surface than the smooth surface within 150 microns from the edge of the PS-device. However, by 200 microns, there were fewer neurons on the nPS side compared to the smooth side.

The effect of surface structure was also studied by comparing mesostructured porous silicon to nanostructured porous silicon as novel surface coatings for ceramic-based microelectrodes [17]. The idea of using nanostructured silicon for a bioactive material was first proposed by [106] due to its ability to induce hydroxyapatite growth. We extended this approach to assess its bioactive properties in the brain. *In vitro* studies showed that neurons preferred the nanostructured surface by extending significantly more neurites while glial cells avoided the nanostructured surfaces, suggesting that this surface may be useful for targeting appropriate cell types *in vivo* (Figure 10). Subsequent *in vivo* studies showed that microelectrodes coated with nanostructured porous silicon could be used to record single neurons [107] (Figure 11) and studies at one week showed less glial activation and more neurons adjacent to porous silicon surfaces than smooth silicon surfaces (Figure 12). More studies over longer time periods are on-going to determine if these surfaces have a useful effect for improving long-term biocompatibility. 

### 4.2. Flexible microelectrodes: Matching the Brain’s Mechanical Properties

Since cells have different responses to materials with different stiffness and there is a potential for injury due to the strain fields caused by the mismatch of mechanical properties between the cells and the microelectrodes several investigators have attempted to build flexible microelectrodes. The major trade-off in such a design is that if the electrode were to match the mechanical properties of the brain, it would be difficult to handle and insert into the brain. Nevertheless, the most popular materials for making flexible microelectrodes are polyimide and Parlylene C [108,109]. To date, these studies generally report that the modulus of elasticity of the resulting microelectrodes are on the order of several GPa [110,111] while the estimated modulus of elasticity of the brain is on the order of kPa [112]. Therefore, it is not clear that these devices are flexible enough to be effective. Moreover, they usually require a guide system in order to ensure that they are properly inserted [113] which may cause excessive damage during implantation. To date, none of these devices have shown any improvement in neuronal recordings compared to conventional materials.

### 4.3 Floating Arrays vs. Untethering the Microelectrode

Another important aspect of the microelectrode that has received limited attention is the impact of tethering the microelectrode to the skull. To date, the signals that are recorded from the microelectrode are transmitted from the tip of the microelectrode to a connector that is fixed to the skull in order to transmit the data for signal processing. It has been suggested that tethering the microelectrode with a flexible cable will allow the microelectrode to ‘float’ in the brain and thereby reduce strain between the brain and the microelectrode [68]. It is hypothesized that the reduction in strain would reduce the upregulation of glia. Two examples of floating microelectrodes is the Michigan probe with integrated silicon ribbon cable [114] and the Utah Array. 

The Michigan Center for Neural Communication Technology developed a silicon ribbon cable that is extremely flexible and can be from 2 to 20 microns in thickness. By using boron doped silicon to define a small (approximately 5 micron diameter) cable between the electrode and the connector, the electrode can essentially move with the brain tissue. The advantage is that the same process that is used to make the electrodes is used to make the cable. Therefore, by applying appropriate masks, the cable and electrode can be integrated during fabrication, eliminating the need to bond the electrode to the cable. This flexible tether allows the microelectrode to ‘float’ in the cortex while recording single neurons. It has been used to record single neurons for up to a year in rats. The silicon ribbon is flexible and deforms with the movement of the brain.

Another approach is the well-known Utah Array. This array consists of up to 100 sharpened needles that project from a silicon substrate. A non-conducting glass electrically isolates the needles and the tips are coated with platinum to make them electrically active recording sites. The tips are typically 50 to 100 microns in length and have electrode impedances from 0.1 to 0.2 megaohms. A pneumatically-actuated impulse inserter implants the array at high velocity into the tissue. The silicon substrate rests on the surface of the brain with the needle-like electrodes are inserted into the tissue. The silicon substrate is connected to a flexible tether that allows the device to ‘float’ on the brain. These devices are now routinely used to record for many months from primates and have recorded for a year in human subjects [2]. The only limitation to these electrodes is that they penetrate a little less than 2 mm into the brain tissue and are, therefore, not appropriate for recording from deep brain structures.

Ideally, however, the tether would be replaced completely with a wireless system [5]. Studies suggest that eliminating the tether altogether does reduce gliosis [115]. The shaft of silicon microelectrodes was either fully implanted into the cortex of rats or tethered with a flexible cable to the scalp. Quantitative analysis of the neural tissue around the microelectrodes up to four weeks after implant suggest that eliminating the tether reduces gliosis and increases neurons around the untethered microelectrode compared to the tethered microelectrodes [115]. The difficulty then is getting power to the electronics on-board the microelectrode that condition the signal and sending the signal telemetrically to a receiver outside the brain. 

Several conductive systems have been proposed that use magnetic waves to transmit power to neural interface devices. For example, a successful device for stimulating peripheral nerves and muscles has been developed using this technology [116,117] however, they are large and not suitable for brain implants. Another approach is to power the signal through optical coupling. For examples [118] have proposed using near-infrared light to transfer energy to an implantable microstimulator. We have been working on a similar concept for recording single neurons. For this device, the design is slightly different. Since we are interested in recording, we require signal conditioning near the microelectrode. Our previous design of a VLSI chip for signal conditioning [119] lays the foundation for interfacing with a composite microelectrode [5] optically. As described above, our nanostrucutred porous silicon microelectrodes have a bioactive surface that minimizes the glial response to microelectrodes and is more conducive to neurite outgrowth than a smooth silicon microelectrode. However, there is a second important reason for using nanostructured porous silicon. It can act like a solar cell.

It was first demonstrated by [120] that nanostructured porous silicon can be photoluminescent at room temperature. This discovery was followed quickly by the observation that it is also electroluminescent, suggesting it is an excellent material for microelectronics [121]. An advantage to nanostructured porous silicon is that its formation can be tuned to minimize reflection [122,123] and increase power generation efficiency. The current commercial goal for most of this work is for improved solar cells. However, it is possible, as we have demonstrated [107] to make thin films of nanostrucutred porous silicon and use them for a microelectrode. The porous silicon can then be used to convert a light source, supplied by a fiber optic cable inserted into the brain tissue near the microelectrode, into power for on-board signal conditioning and telemetry. An additional advantage of nanostrucutred porous silicon is that it appears to be as efficient as other materials used for photodiodes such as gallium arsenic (GaAs) which would not be biocompatible. Moreover, because nanostructured porous silicon can be made with similar thin film processing of very-large-scale integration, unlike GaAs semiconductors, there is a potential to create devices on the order of a few microns [124]. Computational models of such a device demonstrate that within the current size of a microelectrode, a nanostructured porous silicon photodiode could convert light to enough energy to power an 8-channel device. Therefore, if the device needed to be implanted deep into neural tissue, which appears likely for many applications, one could implant the microelectrode and pass a fiber optic cable near the device. The device could be powered using near-infrared light, which passes easily through the tissue [118].

## 5. Conclusions

When attempting to increase the longevity of microelectrode recordings, it is important to recognize that glia are an important part of the process and need to managed, not eliminated. Therefore, approaches utilizing bioactive interventions that attempt to alter to glial response andattract neurons to the recording site are likely to be the most successful. Importantly, measures of the glial scar alone are not sufficient to assess the effect of interventions. It is imperative that recordings of single neurons accompany any study of glial activation because, at this time, we do not know the precise relationship between glial activation and loss of neuronal recordings. Moreover, it is important to have quantitative measures of glial upregulation and neuronal activity in order to assess the relationship between the two. These types of studies will help rationalize the study of interventions to improve the longevity of recordings from microelectrodes.
